# Appearance of L90I and N205S Mutations in Effector Domain of NS1 Gene of pdm (09) H1N1 Virus from India during 2009–2013

**DOI:** 10.1155/2014/861709

**Published:** 2014-09-15

**Authors:** Sachin Kumar, Shashi Khare, Bano Saidullah, Inderjeet Gandhoke, Hanu Ram, Supriya Singh, L. S. Chauhan, Arvind Rai

**Affiliations:** ^1^Division of Microbiology, National Centre for Disease Control, 22 Sham Nath Marg, Delhi 110054, India; ^2^Division of Biotechnology, National Centre for Disease Control, 22 Sham Nath Marg, Delhi 110054, India; ^3^Discipline of Life Science, School of Science, Indira Gandhi National Open University, Delhi 110068, India

## Abstract

In the present study, full length sequencing of NS gene was done in 91 samples which were obtained from patients over the time period of five years from 2009 to 2013. The sequencing of NS gene was undertaken in order to determine the changes/mutations taking place in the NS gene of A H1N1 pdm (09) since its emergence in 2009. Analysis has shown that the majority of samples belong to New York (G1 type) strain with valine at position 123. Effector domain of NS1 protein displays the appearance of three mutations L90I, I123V, and N205S in almost all the samples from 2010 onwards. Phylogenetic analysis of available NS1 sequences from India has grouped all the sequences into four clusters with mean genetic distance ranging from 12% to 24% between the clusters. Variability in length of NS1 protein was seen in sequences from these clusters, 230-amino-acid-residue NS1 for all strains from year 2007 to 2008 and for 21 strains from year 2009 and 219-residue products for 37 strains from year 2009 and all strains from year 2010 to 2013. Mutations like K62R, K131Q, L147R, and A202P were observed for the first time in NS1 protein and their function remains to be determined.

## 1. Introduction

Influenza viruses are responsible for acute respiratory infection and are a source of seasonal epidemics and occasional pandemics. Influenza A viruses are classified into subtypes based on the different types of HA and NA combinations that occur. So far 18 hemagglutinin (HA) and 11 neuraminidase (NA) subtypes have been reported from various organisms ranging between aquatic, avian, and human species [1, 2]. Segment 8 of influenza A (H1N1) encodes two proteins NS1 (nonstructural) protein and NEP (nuclear export protein) by alternative splicing. The mRNAs of both proteins share 56 nucleotides at the 5′ end, resulting in both proteins sharing 10 amino acids at N terminal.

NS1 protein is encoded by the collinear mRNA from segment 8 of the influenza virus genome and has a strain specific length ranging from 230 to 237 amino acid residues. It is expressed exclusively in the infected cells [3]. NS1 could be divided into two functional domains: (i) N-terminal RNA binding domain (residues 1–73) and (ii) C-terminal effector domain, interacting with several host factors (residues 74–230) [3–6].

NS1 is a multifunctional protein involved in various functions of regulating immune responses. It functions as an interferon (IFN) antagonist, which allows efficient virus replication in IFN-competent hosts. NS1 targets both IFN-*α*/*β* production and the activation of IFN-induced antiviral genes [6]. The RNA binding domain (RBD) of NS1 binds to both ssRNA and dsRNA, thereby sequestering them and preventing their recognition by RIG1 (retinoic acid inducible gene), resulting in inhibition of IFN *α* and *β* expression [7, 8]. NS1 protein is also involved in inhibiting 3′ end processing of host mRNA by binding to CPSF 30 (cleavage and polyadenylation specificity factor 30) and PABPN1 (poly(A) binding protein nuclear 1) [9]. Sequestering of dsRNA by RBD of NS1 from 2′–5′ oligoadenylate synthetase (OAS) is essential for inhibition of ribonuclease L (RNase L) pathway, which is involved in the degradation of viral RNA. NS1 binds directly to the regulatory subunit of protein kinase R (PKR) and therefore regulates the effectors of IFN response and controls apoptosis, cell growth, cell proliferation, cytokine production, and signaling [10]. NS1 interacts with eIF4GI and PABP1 (poly(A) binding protein 1) and enhances viral protein synthesis in comparison to host cell protein. In this way, NS1 inhibits the innate immune response of the host by suppressing the interferon release. It also inhibits adaptive immunity by restricting human dendritic cells maturation and induction of T-cell response [11].

NS2 (NEP) is involved in the export of viral RNP from the nucleus to the cytoplasm through nuclear export signal and via interaction with Crm1 protein. NEP can be divided into a protease-sensitive N-terminal domain (amino acids 1–53) and a protease-resistant C-terminal domain (amino acids 54–121) [12]. Of the two domains N-terminal domain has been reported to contain nuclear export signal between residues 12 and 21 which interact with the nuclear export protein Crm1 and facilitate the export of viral RNPs [13].

In the present study, full length sequencing of pdm H1N1 (09) virus for NS gene was performed in samples collected from years 2009 to 2013 in order to determine the mutations taking place in the NS gene of pdm H1N1 (09) virus since its emergence in year 2009. Genetic and phylogenetic analyses of previously studied sequences reported from India and other countries were done, based on available literature in order to determine their phylogeny and sites under selection pressure (contributing towards the evolution of virus) and to study the possible effect of mutations on virulence and pathogenicity of influenza virus.

## 2. Materials and Methods

Samples (Nasal and Throat Swabs in viral transport media (VTM)) from years 2009 to 2013 (details given in Table 1) from patients with symptoms of fever, cough, sore throat, nasal catarrh, or shortness of breath were collected from hospitals of Delhi and outbreak samples from other states obtained for H1N1 testing at the National Centre for Disease Control (NCDC), New Delhi, India. The study was approved by the institutional ethical committee and all the samples were processed in a high containment facility (a biosafety level-3 laboratory) at NCDC, New Delhi. Viral RNA was extracted using QIAmp viral RNA mini kit (Qiagen, Germany) according to manufacturer's protocol. Finally RNA was eluted in 50 *μ*L of elution buffer and stored at −80°C until use. The initial detection of influenza viruses was done by RT PCR protocol for detection of influenza A (H1N1) pdm (09) by WHO/CDC [14, 15].

For sequencing, viral genes were amplified as described earlier [16, 17]. Nucleotide (nt) sequencing was carried out on Applied Biosystems 3130xl Genetic Analyzer (Applied Biosystems, Foster City, CA, USA), using gene specific primers. Nucleotide and protein sequence BLAST (Basic Local Alignment Search Tool) search was performed using the National Centre for Biotechnology Information (NCBI), National Institute of Health, Bethesda, MD, BLAST server at GenBank database [18]. Sequences for phylogenetic analyses were retrieved and multiple sequence alignments were performed on the Influenza Virus Resource (IVR) at NCBI and the Influenza Research Database (IRD) at http://www.fludb.org/ [19, 20]. Phylogenetic analysis was done by MEGA v6.0, using maximum likelihood method and 500-replicate bootstrapping. Mean genetic distance within the cluster and between the clusters was determined by MEGA v6.0 [21].

Metadata-driven comparative analysis of study samples against all protein sequences in the influenza virus database at the Influenza Research Database (IRD) at http://www.fludb.org/ for NS1 protein till 21 December 2013 was performed by Meta-CATS tool [22] on the Influenza Research Database (IRD) at http://www.fludb.org/ at *P* value threshold of 0.05 (*P* value threshold is used as the maximum probability level for the likelihood that the position is different among the groups simply by chance) in order to identify significantly different sites between group 1 (database sequences) and group 2 of the study samples.

Selection pressure analysis acting on the codons of NS (nonstructural) gene of H1N1 pdm virus was carried out using HyPhy open-source software package available under the datamonkey web server (http://www.datamonkey.org/) [23]. Analysis was performed using reference sequences [*n* = 72 (NS)] including Indian H1N1 pdm virus. A separate analysis for NS1 and NEP genes was also carried out by including 44 Indian H1N1 pdm viruses. The ratio of nonsynonymous (dN) to synonymous (dS) substitutions per site (dN/dS or v) was estimated using five different approaches, including single likelihood ancestor counting (SLAC), fixed effects likelihood (FEL), random effects method (REL), mixed effects model of evolution (MEME), and fast unbiased Bayesian approximation (FUBAR). The best nucleotide substitutions model for different data sets as determined through the available tool in datamonkey server was adopted in the analysis.

## 3. Results

### 3.1. Mutations Seen in the NS1 Gene of Influenza A H1N1 pdm (09)

Total 48 nucleotide substitutions (27 synonymous and 21 nonsynonymous) were observed in 91 samples from the years ranging from 2009 to 2013 when compared with FJ969528 (A/California/07/2009) as shown in Tables 2 and 3. I123V mutation was seen in 88 samples, along with other common amino acid changes like E55Q, L90I, and N205S (30–50% samples). Changes like D53N, K62R, S73T, T94A, E96K, R108K, I111T, V129I, V129A, K131E, T143N, I145V, L147R, T151P, E172 K, A202P, and N209D were rare and observed only in few samples as given in Tables 2 and 3.

Mutations I43N, D53N, T94A, R108K, and E172K were observed only in 1–4 samples from year 2009, while two samples had mutation N209D and three had E55Q mutation similar to year 2010. E55Q mutation was also observed in a varied number of samples (given in Tables 2 and 3) from all the years except 2012. Mutation V129A was observed in a single sample each year from 2009 and 2011. Except three samples (two from 2009 and one from 2010) all other 88 samples had I123V mutation in NS1 protein.

Mutations E96K (2 samples) and I145L and T151P (1 sample each) were detected only in samples from 2010. E55Q mutation was the second most common amino acid change after I123V and was seen in 16 samples of year 2010, while three mutations K62K, S73T, and I145V were common among 2010 and 2011 samples (given in Tables 2 and 3).

Mutations V129I (6 samples) and L147R (1 sample) were observed only in samples from 2011. Other mutations observed in a few samples in 2011 were E55Q (6 samples) and S73T (3 samples), while mutations K62R and I145V were found in single samples. The most common amino acid changes among 2011 samples were L90I (8 samples) and N205S (13 samples) which were noted for the first time in the samples of 2011 and then persisted thereon.

Study samples of 2012 were almost like the 2011 samples but a single difference at position K131Q was noted in 2 samples. In samples of the year 2013, a single sample had E55Q mutation which was similar to the mutation observed in 2010 samples. Mutations E55K, E55Q, and A202P in single samples and K131E in 4 samples were only detected in samples of the year 2013.

### 3.2. Mutations in RNA Binding Domain of NS1 Protein (Residues 1–73)

The RNA binding domain is involved in binding and sequestering dsRNA from its recognition by RIG1 and OAS and thereby inhibiting the IFN response against the virus. In samples from 2009 to 2013, synonymous mutations were seen at ten nucleotide positions (14, 18, 27, 31, 36, 38, 44, 53, 68, and 71) and nonsynonymous mutations were noticed at positions I43N, D53N, E55Q, E55Q, K62R, and S73T as given in Table 2. Among these E55Q was the most common change found in 3 samples from 2009, most samples of 2010 (16), 6 samples from 2011, and 1 sample from 2013. Other changes were rare and only seen in two or three samples.

### 3.3. Mutations in Effector Domain of NS1 Gene

In total 17 synonymous mutations were observed at the following positions 83, 85, 88, 99, 105, 125, 132, 138, 142, 143, 144, 152, 153, 163, 186, 214, and 217 from the year 2009 to 2013, while 17 nonsynonymous mutations were recognized at positions L90I, T94A, E96K, R108K, I111T, I123V, V129I, V129A, K131E, T143N, I145V, L147R, T151P, E172K, A202P, N205S, and N209D (in Table 2). I123V was the most common mutation seen in about 97% of samples, followed by E55Q, L90I, and N205S mutations which were noted in about 30% to 50% of samples. The remaining mutations were seen in 1% to 6% samples.

Metadata-driven comparative analysis tool (meta-CATS) of NS1 protein sequence between all database sequences and study sample sequences was performed for identification of amino acid positions that significantly differ between two or more groups of virus sequences. A total 79 sites were identified by Meta-CATS as sites having a significant nonrandom distribution between the specified groups (database sequences and study sequences). 18 of 79 sites identified by Meta-CATS were similar to sites with amino acid changes in study samples and most of the changes seen in the samples were common to sequences in the database. However, mutations like E96K and V129A were rare and viewed only in a limited number of samples in the database, while changes like K62R and K131Q were unique and seen only in one or two study samples.

### 3.4. Mutations in NEP Gene

NEP is reported to be involved in nuclear export of viral ribonucleoprotein (RNP) complexes and is conserved in comparison to NS1. Seven synonymous and 4 nonsynonymous mutations were observed in NEP gene from year 2009 to 2013. Amino acid changes noticed in NEP were M14I, N29S, T48A, and S60N among which T48A is the most common change found in around 50% of samples. Other mutations seen were M14I (2 samples) from year 2009, N29S (2 samples) and S60N (7 samples) from year 2013.

Study samples of 2009 and 2010 were similar to A/California/07/2009. However, in two samples from year 2009 single amino acid change replacing methionine at position 14 with isoleucine (M14I) was noticed in nuclear export signal of NEP protein. T48A mutation first appeared in 2011 and persisted thereon. Samples from 2012 were similar to 2011 with no change. Samples from 2013 displayed two mutations: N29S in two samples and S60N in 50% of the samples.

### 3.5. Selection Pressure Analysis

Selection pressure analysis of NS gene of influenza A H1N1 pdm virus strain revealed 8 positively selected sites. Integrated analysis was performed for differential selection pressure acting on NS1 (219 codons) and NEP (121 codons) proteins (shown in Table 4). Out of seven NS1 sites, one was located in RBD and six in ED. Analysis of NEP protein gene revealed single position 49 to be under positive selection. A specific selection pressure analysis for Indian isolates (*n* = 44) for NS1 and (*n* = 21) for NEP gene revealed 3 sites in NS1 and 1 site in NEP gene under positive selection.

### 3.6. Analysis of Available NS1 Sequences from India

**Figure 1:**
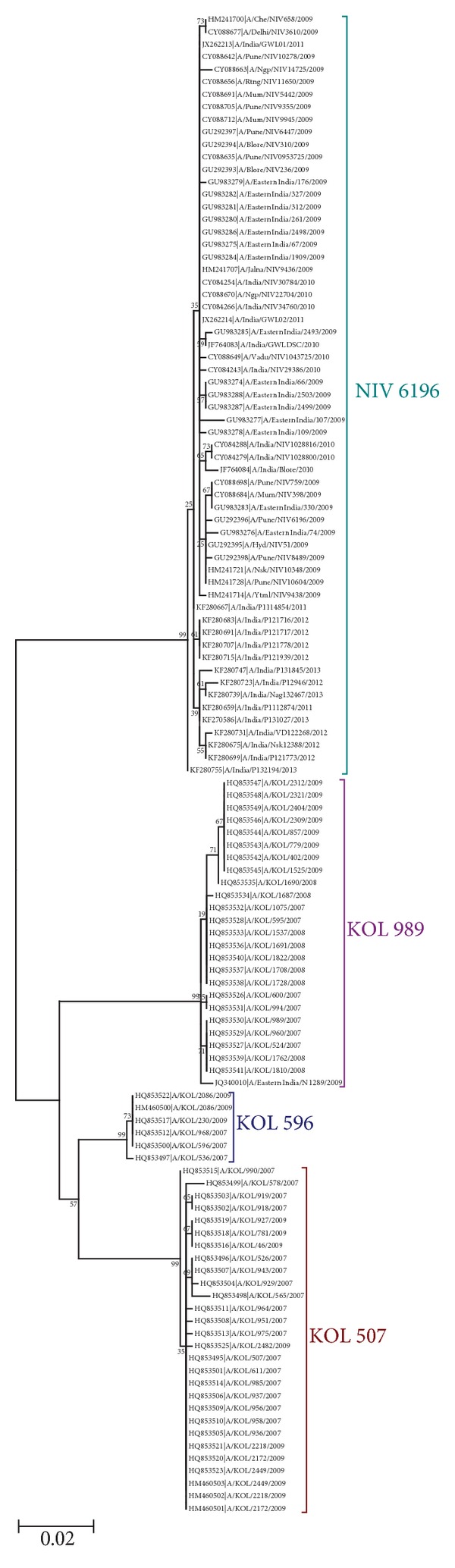
Phylogenetic tree of all sequences (120 sequences from India, 2007–2013) of NS1 gene of influenza A (H1N1) was constructed using maximum likelihood method in MEGA 6.0 software. Bootstrap values at 500 replications were shown at branches. Each node showing accession number with strain name. Phylogenetic tree is depicting 4 major clusters or groups represented by their cluster name given in front of each cluster.

Full length NS1 gene sequences (120 sequences) available from India till 31 March 2014 were retrieved from the Influenza Research Database (IRD) at http://www.fludb.org/. Sequences were phylogenetically analyzed by maximum likelihood method which grouped all sequences into 4 clusters. These clusters were represented by a single representative strain of each cluster from hereon: A/KOL/507/2007 (KOL 507), A/KOL/596/2007 (KOL 596), A/KOL/989/2007 (KOL 989), and A/Pune/NIV 6196/2009 (NIV 6196) (shown in Figure 1). Among these, KOL 507 and KOL 596 clusters had sequences from the years 2007 and 2009, while, in KOL 989 cluster, sequences from year 2007, 2008, and 2009 were seen. NIV 6196 cluster was noted to have sequences from 2009 to 2013. KOL 596 like strains constituted the smallest group with 6 samples, while NIV 6196 like strains formed the largest group with 61 samples.

NS1 protein encoded by clusters KOL 507, KOL 596, and KOL 989 was of 230 amino acid residues in length, whereas NIV 6196 cluster encoding NS1 protein was of 219 amino acid residues. Due to difference in length of NS1 protein, 12 sites were only seen in clusters encoding 230 amino acid residues' NS1 protein. Terminal amino sequence of avian influenza A (H5N1) virus NS1 protein is reported to be associated with virulence and pathogenicity (30). NS1 protein encoded by clusters has different C-terminal amino acid sequence; KOL 507 and KOL 596 have RSEV, KOL 989 had RSKV, and NIV 6196 had PEQK.

Multiple sequence alignment of 120 sequences from 2007 to 2013 strain of all clusters (from India) showed differences in amino acid sequence at 100 sites between the clusters when compared with reference to KOL 507 cluster of which some sites were cluster specific (shown in Table 5), while others were common between clusters (Shown in Table 6).

KOL 507 and KOL 596 clusters have no year specific distribution of mutations or signature sequence within the cluster. KOL 989 cluster have one such pattern in sequences from year 2009, which has arginine at position 135 and glycine at position 139 in place of serine and aspartic acid. The NIV 6196 cluster has isoleucine at position 90 and serine at position 205 in place of leucine and arginine in the majority of the samples from 2011 to 2013.

Mean distance in NS1 protein sequence between clusters with reference to KOL 507 cluster was approximately 12% for KOL 596 cluster, 17% for KOL 989 cluster, and 25% for NIV 6196 cluster. All clusters have maximum sequence dissimilarity of 1% between the sequences within the cluster except NIV 6196 which has the maximum dissimilarity of 2% within the cluster. It has been observed that all study samples (2009–2013) belonged to NIV 6196 cluster and no circulation of strain similar to KOL 507, KOL 596, and KOL 989 like strains has been seen in the last four years.

Mutations in two functional domains of NS1 protein were observed between various clusters which affect their function. RNA binding domain of NS1 protein has mutations at positions 41, 44, and 67 which are involved in binding dsRNA. Mutation at positions 41 and 44 were noticed in KOL 596 and KOL 989 clusters, whereas change at position 67 was seen in clusters KOL 989 and NIV 6196. Effector domain of NS1 protein has mutation at 12 positions: 91, 95, 98, 101, 117, 119, 123, 125, 135, 144, 145, and 145 which may affect its interaction with host protein. Mutations at positions 95, 143, and 145 were seen in all the clusters. Some changes were cluster specific: 98, 135, and 144 in KOL 989 cluster, 91, 119, and 123 in NIV 6196 cluster while others were common between two clusters 101 and 117 in KOL 596 and KOL 989 clusters, 125 in KOL 989 and NIV 6196 clusters.

## 4. Discussion

NS1 protein is responsible for regulation of antiviral immune response in the host cells and a number of NS1 molecular markers are reported to be associated with increased virulence and pathogenicity like R38, F103, and M106 [7, 24]. NS1 protein is functionally divided into two domains: RNA binding domain (RBD) and effector domain (ED). RBD is mainly involved in sequestering of dsRNA from OAS and RIG1. In the present study, analysis of sequencing data from NS1 gene showed relatively conserved RBD in comparison to ED (shown in Table 2). Only five amino acid changes were seen in RBD, out of which E55Q was the most common change in comparison to other mutations which were rare and occurred in two or three samples only. None of the changes occurred in positions reported to be involved in RNA binding [7].

Effector domain is involved in interactions with the host factors, associated with cell signaling and immune response. I123V mutation was seen in ED of almost all study samples (shown in Tables 2 and 3) and was categorized into New York (G1 type) strains [25]. Apart from this mutation, L90I and N205S mutations were found to occur over three years in a large number of samples. Other mutations which were detected in 20% or more samples were R108K (year 2009), I145V (year 2010), V129I (year 2011), and K131E (year 2013). The rest of the mutations were seen to occur only in one or two samples in all years.

Glutamate at position 96 is functionally important for binding of NS1 to CPSF30 and necessary for interaction with TRIM25, a ubiquitin ligase which mediates the ubiquitination of the RIG-1 (a viral RNA sensor) in order to facilitate IFN production. It has been reported that E96A mutants were ineffective in blocking TRIM25 mediated IFN response [8, 26]. E96K substitution was noted in 2 samples from 2010, while the rest of 89 samples have E96, which shows that the majority of viruses in circulation with E96 are competent enough to inhibit TRIM25 mediated immune response and replicate efficiently in host cell.

It has been reported that interaction of NS1 residues 123–127 with PKR results in inhibition of eIF2α phosphorylation and viral protein synthesis, indicating that NS1-PKR binding is necessary and sufficient to block PKR activation in influenza A virus-infected cells [27]. In the present study eighty-eight samples were seen to have I123V mutation in this region. I123V mutation may therefore affect the inactivation of PKR by NS1 protein.

It has also been reported that NS1 protein with R108, E125, and G189 is unable to block the host gene expression resulting in inefficient replication of virus. This inhibitory effect could be restored by replacing above residue with residues corresponding to the human H1N1 virus consensus sequence [28]. One of these mutations R108K was seen in 4 samples from year 2009.

It has been reported that the influenza A (H5N1) NS1 protein interacts with eukaryotic translation initiation factor 4GI (eIF4GI) via eIF4GI binding domain (residues 81–113) resulting in the preferential translation of the viral mRNA in comparison to host mRNA [29]. Therefore, mutation in this domain may result in impaired ability of virus to inhibit interferon production which may result in inefficient virus replication. L90I and T94A mutations may, therefore, affect interferon response and virus replication. Similarly, in ferrets it has been reported that human (H5N1) virus with arginine (N) at position 205 of NS1 protein enhances the type I IFN antagonistic property of the host cell leading to high virulence in ferrets [30]. In the present study samples, we have seen N205S mutation in all samples from 2011 onwards.

In this study the nuclear export signal of NEP displays M14I mutation in 2 samples from 2009, while the C-terminal domain of NEP was reported to interact with the nuclear localization signal of the viral matrix protein M1 [31] which has shown two mutations, T48A in almost all samples from 2011–2013, S60N in 50% of samples from 2013. These mutations may affect nuclear transport and release of virus from cell.

Selection pressure analysis of NS gene of influenza A H1N1 pdm virus strain revealed 8 positively selected sites (shown in Table 4). Positions 108, 123, 145, 147, and 205 were noted to be situated in NS1 protein host factor interaction domains. Analysis of NEP protein gene revealed single position 49 to be under positive selection. A specific selection pressure analysis for Indian isolates revealed 3 sites in NS1 and 1 site in NEP gene to be under positive selection. Positions 55, 129, and 145 in NS1 gene were found to be common between India specific isolates and reference strain isolates. This showed that positive selection on NS1 gene was stronger than that on NEP, of which a large number of sites were located in influenza host factor interaction domains, which are reported to be associated with virulence and pathogenicity of influenza virus [3, 26–29].

**Figure 2:**
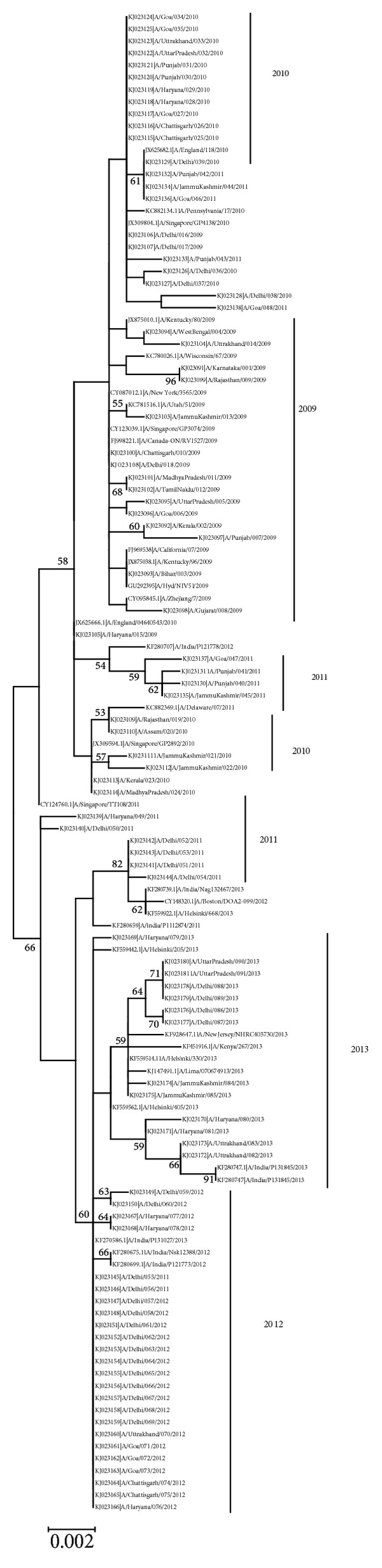
Phylogenetic tree of influenza A (H1N1) virus based on NS1 gene generated by the maximum likelihood method. Bootstrap support values (based on 500 replications) above 50% are shown at the branch node. Each branch is denoted by accession number and strain name.

Phylogenetic analysis of study samples on the basis of NS1 gene of influenza A (H1N1) virus broadly grouped all sequences into two major branches (shown in Figure 2). One (group one) is with samples from year 2009 to 2011 and the other (group two) is with samples from 2011 to 2013. In comparison to reference strain samples for year 2009 were most similar with mean distance of 0.9% followed by mean distance of 1.1% for 2011 samples, 1.7% for 2011 samples, 1.4% for 2012, and 1.6% for 2013 samples. This showed that samples from 2011 were most dissimilar to reference strain, followed by 2013 samples. Phylogenetic analysis showed that samples from year 2009 were almost identical to reference strain (A/California/07/2009). The majority of 2010 samples showed homology with A/Singapore/GP4138/2010 and A/Pennsylvania/17/2010, while 6 strains showed homology with A/Singapore/GP2892/2010 strain. Samples from each year formed a discrete branch on the tree except for samples from year 2011, which were seen in both groups. 2011 samples in group one were seen in three separate branches: one branch with four samples showing homology to A/India/P121778/2012 strain and another with three samples having homology with A/England/118/2010 strain and single samples with homology to A/Singapore/GP4138/2010 strain. However, 2011 samples in group two formed two separate branches: one at base of group two containing two samples and the other with four samples, which showed homology to A/Boston/DOA2-099/2012 strain. Samples from year 2012 showed homology to A/India/Nsk12388/2012 strain, while 2013 samples had homology with A/Helsinki/405/2013 and A/New Jersey/NHRC403730/2013 strains. Mutations I123V and N205S in NS1 protein observed in the present study have also been observed in a large number of sequences from Europe, America, Africa, and Asia. While L90I (NS1 protein) was seen in limited number of sample from Europe, America and Africa. T48A (NEP protein) was seen only in few samples from Europe, Asia, Africa, and America. An earlier study on H1N1 pdm (09) sequences from India involving 13 samples has also reported the mutation reported in this study [32]. However, in comparison to that study, the present study has used a larger number of samples and found additional mutation in NS1 gene.

Phylogenetic analyses of 120 full length NS1 sequences from India during the time period 2007–2013 (retrieved from the Influenza Research Database (IRD) at http://www.fludb.org/) were found to be grouped into four clusters as shown earlier in Figure 1. NS1 protein encoded by KOL 507, KOL 596, and KOL 989 cluster was seen to be of 230 amino acid residues in length, whereas NIV 6196 like strains were seen to encode 219 residue long NS1 protein. Our investigation reveals that influenza A (H1N1) is evolving and acquiring mutations, which could be noted, by observing the mean distance in NS1 protein sequence between the clusters, approximately 12% for KOL 596 cluster, 17% for KOL 989 cluster, and 25% for NIV 6196 cluster. NS1 protein of none of the clusters was seen to have ESEV, EPEV, and KSEV as their terminal amino acid sequence, which are reported to be associated with increased virulence in influenza A (H5N1) virus. All study samples belonged to NIV 6196 cluster and had loss of 11 amino acids at c-terminal end of NS1 protein. Analysis of NS1 protein shows that the four clusters were derived from three major reassortment events, with KOL 989 cluster derived from seasonal H3N2 virus, KOL 507 and KOL 596 clusters from prepandemic seasonal H1N1, and NIV 6196 cluster from H1N1 pdm (09) lineage. This has resulted in large mean distance between cluster and loss of terminal amino acid residue. High values for mean distance and loss of residue between NIV 619 cluster and KOL 507 could be explained by introduction of pandemic strain in year 2009 in human population. Circulation of three different clusters in period of four years from 2007 to 2009, with high mean distance between them, shows that influenza A virus has evolved rapidly (by antigenic shift and drift) in the past and it could do so in the future, which highlights the need of continuing surveillance and monitoring of influenza virus infection across the nation and worldwide.

## 5. Conclusions

Sequence analysis shows that NS1 protein is mutating more rapidly than NEP and that within NS1 protein RBD is more conserved than ED. The prominent change seen in RBD was E55Q in samples from 2009 to 2011. Within RBD, no change has been seen in sites reported to be involved in RNA binding, inhibiting IFN responses, and which are therefore believed to be efficient in sequestering dsRNA and inhibiting antiviral responses. Study of effector domain has displayed a number of changes in sites/domains reported to be associated with host factor interaction. Three major mutations identified in the ED were I123V which was seen in almost all samples from 2009 to 2013, while L90I and N205S mutations were found for the first time in samples from 2011 and then persisted onwards. It has been observed that the majority of the study samples were of New York (G1 type) with valine at position 123. Most of the mutations in the sequences observed in this study have also been reported from Asia, Europe, and America. Available NS1 sequences from India show that NS1 is evolving and acquiring mutations with the loss of terminal amino acid residues. On the basis of sequence similarity and available literature, we can say that the present circulating NS1 protein is an effective interferon antagonist. Mutations like K62R, K131Q, L147R, and A202P were seen for the first time in NS1 protein and their effect is yet to be determined.

## Figures and Tables

**Table 1:** Details of patients with strain name, collection date, and genes sequenced per strain with accession number.

S. number	Strain name	Collection date	Sex	Age (years)	Accession number
1	A/Karnataka/001/2009	6-Aug-2009	F	21	KJ023091
2	A/Kerala/002/2009	14-Aug-2009	M	36	KJ023092
3	A/Bihar/003/2009	20-Aug-2009	M	5	KJ023093
4	A/West Bengal/004/2009	6-Dec-2009	F	4.6	KJ023094
5	A/Uttar Pradesh/005/2009	4-Oct-2009	F	17	KJ023095
6	A/Goa/006/2009	6-Oct-2009	M	47	KJ023096
7	A/Punjab/007/2009	26-Nov-2009	M	6	KJ023097
8	A/Gujarat/008/2009	8-Aug-2009	M	0.9	KJ023098
9	A/Rajasthan/009/2009	26-Nov-2009	M	1.6	KJ023099
10	A/Chhattisgarh/010/2009	30-Sep-2009	M	30	KJ023100
11	A/Madhya Pradesh/011/2009	10-Oct-2009	F	55	KJ023101
12	A/Tamil Naidu/012/2009	6-Nov-2009	M	15	KJ023102
13	A/Jammu Kashmir/013/2009	4-Dec-2009	M	14	KJ023103
14	A/Uttarakhand/014/2009	8-Dec-2009	M	5	KJ023104
15	A/Haryana/015/2009	16-Dec-2009	M	60	KJ023105
16	A/Delhi/016/2009	14-Dec-2009	F	6.6	KJ023106
17	A/Delhi/017/2009	13-Dec-2009	M	25	KJ023107
18	A/Delhi/018/2009	29-Jul-2009	F	74	KJ023108
19	A/Rajasthan/019/2010	10-Jan-2010	M	19	KJ023109
20	A/Assam/020/2010	28-Jan-2010	F	33	KJ023110
21	A/Jammu Kashmir/021/2010	19-Jul-2010	M	14	KJ023111
22	A/Jammu Kashmir/022/2010	15-Jan-2010	F	60	KJ023112
23	A/Kerala/023/2010	19-Jun-2010	F	21	KJ023113
24	A/Madhya Pradesh/024/2010	28-Jan-2010	M	47	KJ023114
25	A/Chhattisgarh/025/2010	7-Feb-2010	M	22	KJ023115
26	A/Chhattisgarh/026/2010	25-Sep-2010	F	30	KJ023116
27	A/Goa/027/2010	26-Aug-2010	M	33	KJ023117
28	A/Haryana/028/2010	19-Oct-2010	M	0.6	KJ023118
29	A/Haryana/029/2010	15-Aug-2010	M	56	KJ023119
30	A/Punjab/030/2010	21-Aug-2010	F	35	KJ023120
31	A/Punjab/031/2010	19-Oct-2010	F	4.6	KJ023121
32	A/Uttar Pradesh/032/2010	16-Sep-2010	M	27	KJ023122
33	A/Uttarakhand/033/2010	24-Sep-2010	M	2.6	KJ023123
34	A/Goa/034/2010	7-Aug-2010	M	32	KJ023124
35	A/Goa/035/2010	9-Oct-2010	F	35	KJ023125
36	A/Delhi/036/2010	20-Aug-2010	M	50	KJ023126
37	A/Delhi/037/2010	25-Aug-2010	M	18	KJ023127
38	A/Delhi/038/2010	22-Aug-2010	F	25	KJ023128
39	A/Delhi/039/2010	17-Sep-2010	M	14	KJ023129
40	A/Punjab/040/2011	21-Feb-2011	M	40	KJ023130
41	A/Punjab/041/2011	9-Mar-2011	F	20	KJ023131
42	A/Punjab/042/2011	17-Mar-2011	F	55	KJ023132
43	A/Punjab/043/2011	28-Mar-2011	F	27	KJ023133
44	A/Jammu Kashmir/044/2011	20-Jan-2011	F	25	KJ023134
45	A/Jammu Kashmir/045/2011	31-Jan-2011	M	40	KJ023135
46	A/Goa/046/2011	23-Jun-2011	M	60	KJ023136
47	A/Goa/047/2011	7-Jun-2011	F	30	KJ023137
48	A/Goa/048/2011	19-May-2011	M	18	KJ023138
49	A/Haryana/049/2011	24-Mar-2011	M	19	KJ023139
50	A/Delhi/050/2011	19-May-2011	F	35	KJ023140
51	A/Delhi/051/2011	24-Feb-2011	F	36	KJ023141
52	A/Delhi/052/2011	24-Feb-2011	M	12	KJ023142
53	A/Delhi/053/2011	23-Jun-2011	M	10	KJ023143
54	A/Delhi/054/2011	1-Apr-2011	F	25	KJ023144
55	A/Delhi/055/2011	3-Apr-2011	F	16	KJ023145
56	A/Delhi/056/2011	5-Mar-2011	M	36	KJ023146
57	A/Delhi/057/2012	26-Jul-2012	M	3.6	KJ023147
58	A/Delhi/058/2012	14-Nov-2012	M	3	KJ023148
59	A/Delhi/059/2012	9-Oct-2012	M	34	KJ023149
60	A/Delhi/060/2012	13-Sep-2012	M	28	KJ023150
61	A/Delhi/061/2012	21-May-2012	F	58	KJ023151
62	A/Delhi/062/2012	16-Aug-2012	M	48	KJ023152
63	A/Delhi/063/2012	25-Sep-2012	M	32	KJ023153
64	A/Delhi/064/2012	10-Mar-2012	M	22	KJ023154
65	A/Delhi/065/2012	9-Mar-2012	F	25	KJ023155
66	A/Delhi/066/2012	24-Sep-2012	F	3	KJ023156
67	A/Delhi/067/2012	25-Sep-2012	M	32	KJ023157
68	A/Delhi/068/2012	20-Nov-2012	M	65	KJ023158
69	A/Delhi/069/2012	19-Dec-2012	M	48	KJ023159
70	A/Uttarakhand/070/2012	4-Oct-2012	M	26	KJ023160
71	A/Goa/071/2012	15-May-2012	F	60	KJ023161
72	A/Goa/072/2012	9-Oct-2012	F	40	KJ023162
73	A/Goa/073/2012	9-Jan-2012	M	20	KJ023163
74	A/Chhattisgarh/074/2012	10-May-2012	M	62	KJ023164
75	A/Chhattisgarh/075/2012	10-Aug-2012	F	21	KJ023165
76	A/Haryana/076/2012	11-Jun-2012	M	55	KJ023166
77	A/Haryana/077/2012	20-Nov-2012	F	24	KJ023167
78	A/Haryana/078/2012	17-Feb-2012	F	42	KJ023168
79	A/Haryana/079/2013	2-Jan-2013	F	75	KJ023169
80	A/Haryana/080/2013	2-May-2013	M	52	KJ023170
81	A/Haryana/081/2013	22-Jan-2013	M	70	KJ023171
82	A/Uttarakhand/082/2013	18-Feb-2013	F	47	KJ023172
83	A/Uttarakhand/083/2013	22-Feb-2013	F	30	KJ023173
84	A/Jammu Kashmir/084/2013	24-Feb-2013	M	52	KJ023174
85	A/Jammu Kashmir/085/2013	20-Feb-2013	F	31	KJ023175
86	A/Delhi/086/2013	31-Jan-2013	F	60	KJ023176
87	A/Delhi/087/2013	17-Feb-2013	M	13	KJ023177
88	A/Delhi/088/2013	18-Feb-2013	F	2	KJ023178
89	A/Delhi/089/2013	22-Feb-2013	F	55	KJ023179
90	A/Uttar Pradesh/090/2013	26-Feb-2013	F	25	KJ023180
91	A/Uttar Pradesh/091/2013	28-Feb-2013	M	62	KJ023181

**Table 2:** Year-wise samples showing amino acid changes in different domains of NS1 gene.

Domain	Position	Prevalence of mutation in samples each year				
		2009 (18)	2010 (21)	2011 (17)	2012 (22)	2013 (13)
RNA binding domain	I 43 N	1	—	—	—	—
	D 53 N	2	—	—	—	—
	E 55 Q	3	16	6	—	1
	E 55 K	—	—	—	—	1
	K 62 R	—	1	1	—	—
	S 73 T	—	1	3	—	—

Effector domain	L 90 I	—	—	8	all	all
	T 94 A	2	—	—	—	—
	E 96 K	—	2	—	—	—
	R 108 K	4	—	—	—	—
	I 123 V	16	20	all	all	all
	V 129 A	1	—	1	—	—
	V 129 I	—	—	6	—	—
	K 131 E	—	—	—	—	4
	K 131 Q	—	—	—	2	—
	I 145 L	—	1	—	—	—
	I 145 V	—	5	1	—	—
	L 147 R	—	—	1	—	—
	T 151 P	—	1	—	—	—
	E 172 K	2	—	—	—	—
	A 202 P	—	—	—	—	1
	N 205 S	—	—	13	all	all
	N 209 D	2	1	—	—	—

**Table 3:** NS1 protein positions with variable amino acids in study samples (2009–2013) with reference to A/California/07/2009 reference strain for pandemic H1N1 2009. Reference strain sequence is highlighted with bold font and study samples were arranged according to year of collection and separated with horizontal line.

	53	55	62	73	90	94	96	108	111	123	129	131	143	145	147	151	172	202	205	209
A/California/07/2009 (reference)	D	E	K	S	L	T	E	R	I	I	V	K	T	I	L	T	E	A	N	N

A/Karnataka/001/2009	N	*·*	*·*	*·*	*·*	*·*	*·*	K	*·*	V	*·*	*·*	N	*·*	*·*	*·*	*·*	*·*	*·*	D
A/Kerala/002/2009	*·*	*·*	*·*	*·*	*·*	A	*·*	*·*	*·*	V	*·*	*·*	*·*	*·*	*·*	*·*	K	*·*	*·*	*·*
A/Bihar/003/2009	*·*	*·*	*·*	*·*	*·*	*·*	*·*	*·*	*·*	*·*	*·*	*·*	*·*	*·*	*·*	*·*	*·*	*·*	*·*	*·*
A/West Bengal/004/2009	*·*	*·*	*·*	*·*	*·*	*·*	*·*	K	*·*	V	*·*	*·*	*·*	*·*	*·*	*·*	*·*	*·*	*·*	*·*
A/Uttar Pradesh/005/2009	*·*	*·*	*·*	*·*	*·*	*·*	*·*	*·*	*·*	*·*	*·*	*·*	*·*	*·*	*·*	*·*	*·*	*·*	*·*	*·*
A/Goa/006/2009	*·*	*·*	*·*	*·*	*·*	*·*	*·*	*·*	*·*	V	*·*	*·*	*·*	*·*	*·*	*·*	*·*	*·*	*·*	*·*
A/Punjab/007/2009	*·*	*·*	*·*	*·*	*·*	A	*·*	*·*	*·*	V	*·*	*·*	*·*	*·*	*·*	*·*	K	*·*	*·*	*·*
A/Gujarat/008/2009	*·*	*·*	*·*	*·*	*·*	*·*	*·*	*·*	*·*	V	*·*	*·*	*·*	*·*	*·*	*·*	*·*	*·*	*·*	*·*
A/Rajasthan/009/2009	N	*·*	*·*	*·*	*·*	*·*	*·*	K	*·*	V	*·*	*·*	N	*·*	*·*	*·*	*·*	*·*	*·*	D
A/Chhattisgarh/010/2009	*·*	*·*	*·*	*·*	*·*	*·*	*·*	*·*	*·*	V	*·*	*·*	*·*	*·*	*·*	*·*	*·*	*·*	*·*	*·*
A/Madhya Pradesh/011/2009	*·*	*·*	*·*	*·*	*·*	*·*	*·*	*·*	*·*	V	*·*	*·*	*·*	*·*	*·*	*·*	*·*	*·*	*·*	*·*
A/Tamil Naidu/012/2009	*·*	*·*	*·*	*·*	*·*	*·*	*·*	*·*	*·*	V	*·*	*·*	*·*	*·*	*·*	*·*	*·*	*·*	*·*	*·*
A/Jammu Kashmir/013/2009	*·*	*·*	*·*	*·*	*·*	*·*	*·*	*·*	*·*	V	A	*·*	*·*	*·*	*·*	*·*	*·*	*·*	*·*	*·*
A/Uttarakhand/014/2009	*·*	Q	*·*	*·*	*·*	*·*	*·*	K	*·*	V	*·*	*·*	*·*	*·*	*·*	*·*	*·*	*·*	*·*	*·*
A/Haryana/015/2009	*·*	*·*	*·*	*·*	*·*	*·*	*·*	*·*	*·*	V	*·*	*·*	*·*	*·*	*·*	*·*	*·*	*·*	*·*	*·*
A/Delhi/016/2009	*·*	Q	*·*	*·*	*·*	*·*	*·*	*·*	*·*	V	*·*	*·*	*·*	*·*	*·*	*·*	*·*	*·*	*·*	*·*
A/Delhi/017/2009	*·*	Q	*·*	*·*	*·*	*·*	*·*	*·*	*·*	V	*·*	*·*	*·*	*·*	*·*	*·*	*·*	*·*	*·*	*·*
A/Delhi/018/2009	*·*	*·*	*·*	*·*	*·*	*·*	*·*	*·*	*·*	V	*·*	*·*	*·*	*·*	*·*	*·*	*·*	*·*	*·*	*·*

A/Rajasthan/019/2010	*·*	*·*	*·*	*·*	*·*	*·*	*·*	*·*	*·*	V	*·*	*·*	*·*	V	*·*	*·*	*·*	*·*	*·*	*·*
A/Assam/020/2010	*·*	*·*	*·*	*·*	*·*	*·*	*·*	*·*	*·*	V	*·*	*·*	*·*	V	*·*	*·*	*·*	*·*	*·*	*·*
A/Jammu Kashmir/021/2010	*·*	*·*	*·*	*·*	*·*	*·*	K	*·*	*·*	V	*·*	*·*	*·*	L	*·*	*·*	*·*	*·*	*·*	*·*
A/Jammu Kashmir/022/2010	*·*	Q	*·*	*·*	*·*	*·*	K	*·*	*·*	V	*·*	*·*	*·*	V	*·*	*·*	*·*	*·*	*·*	*·*
A/Kerala/023/2010	*·*	*·*	*·*	*·*	*·*	*·*	*·*	*·*	*·*	V	*·*	*·*	*·*	V	*·*	*·*	*·*	*·*	*·*	*·*
A/Madhya Pradesh/024/2010	*·*	*·*	*·*	*·*	*·*	*·*	*·*	*·*	*·*	V	*·*	*·*	*·*	V	*·*	*·*	*·*	*·*	*·*	*·*
A/Chhattisgarh/025/2010	*·*	Q	*·*	*·*	*·*	*·*	*·*	*·*	*·*	V	*·*	*·*	*·*	*·*	*·*	*·*	*·*	*·*	*·*	*·*
A/Chhattisgarh/026/2010	*·*	Q	*·*	*·*	*·*	*·*	*·*	*·*	*·*	V	*·*	*·*	*·*	*·*	*·*	*·*	*·*	*·*	*·*	*·*
A/Goa/027/2010	*·*	Q	*·*	*·*	*·*	*·*	*·*	*·*	*·*	V	*·*	*·*	*·*	*·*	*·*	*·*	*·*	*·*	*·*	*·*
A/Haryana/028/2010	*·*	Q	*·*	*·*	*·*	*·*	*·*	*·*	*·*	V	*·*	*·*	*·*	*·*	*·*	*·*	*·*	*·*	*·*	*·*
A/Haryana/029/2010	*·*	Q	*·*	*·*	*·*	*·*	*·*	*·*	*·*	V	*·*	*·*	*·*	*·*	*·*	*·*	*·*	*·*	*·*	*·*
A/Punjab/030/2010	*·*	Q	*·*	*·*	*·*	*·*	*·*	*·*	*·*	V	*·*	*·*	*·*	*·*	*·*	*·*	*·*	*·*	*·*	*·*
A/Punjab/031/2010	*·*	Q	*·*	*·*	*·*	*·*	*·*	*·*	*·*	V	*·*	*·*	*·*	*·*	*·*	*·*	*·*	*·*	*·*	*·*
A/Uttar Pradesh/032/2010	*·*	Q	*·*	*·*	*·*	*·*	*·*	*·*	*·*	V	*·*	*·*	*·*	*·*	*·*	*·*	*·*	*·*	*·*	*·*
A/Uttarakhand/033/2010	*·*	Q	*·*	*·*	*·*	*·*	*·*	*·*	*·*	V	*·*	*·*	*·*	*·*	*·*	*·*	*·*	*·*	*·*	*·*
A/Goa/034/2010	*·*	Q	*·*	*·*	*·*	*·*	*·*	*·*	*·*	V	*·*	*·*	*·*	*·*	*·*	*·*	*·*	*·*	*·*	*·*
A/Goa/035/2010	*·*	Q	*·*	*·*	*·*	*·*	*·*	*·*	*·*	V	*·*	*·*	*·*	*·*	*·*	*·*	*·*	*·*	*·*	*·*
A/Delhi/036/2010	*·*	Q	*·*	*·*	*·*	*·*	*·*	*·*	*·*	V	*·*	*·*	*·*	*·*	*·*	*·*	*·*	*·*	*·*	*·*
A/Delhi/037/2010	*·*	Q	*·*	*·*	*·*	*·*	*·*	*·*	*·*	V	*·*	*·*	*·*	*·*	*·*	*·*	*·*	*·*	*·*	*·*
A/Delhi/038/2010	*·*	Q	R	*·*	*·*	*·*	*·*	*·*	*·*	*·*	*·*	*·*	*·*	*·*	*·*	P	*·*	*·*	*·*	D
A/Delhi/039/2010	*·*	Q	*·*	T	*·*	*·*	*·*	*·*	*·*	V	*·*	*·*	*·*	*·*	*·*	*·*	*·*	*·*	*·*	*·*

A/Punjab/040/2011	*·*	*·*	*·*	*·*	*·*	*·*	*·*	*·*	*·*	V	I	*·*	*·*	*·*	R	*·*	*·*	*·*	S	*·*
A/Punjab/041/2011	*·*	*·*	*·*	*·*	*·*	*·*	*·*	*·*	*·*	V	I	*·*	*·*	*·*	*·*	*·*	*·*	*·*	S	*·*
A/Punjab/042/2011	*·*	Q	*·*	T	*·*	*·*	*·*	*·*	*·*	V	*·*	*·*	*·*	*·*	*·*	*·*	*·*	*·*	*·*	*·*
A/Punjab/043/2011	*·*	Q	*·*	*·*	*·*	*·*	*·*	*·*	*·*	V	A	*·*	*·*	*·*	*·*	*·*	*·*	*·*	*·*	*·*
A/Jammu Kashmir/044/2011	*·*	Q	*·*	T	*·*	*·*	*·*	*·*	*·*	V	*·*	*·*	*·*	*·*	*·*	*·*	*·*	*·*	*·*	*·*
A/Jammu Kashmir/045/2011	*·*	*·*	*·*	*·*	*·*	*·*	*·*	*·*	*·*	V	I	*·*	*·*	*·*	*·*	*·*	*·*	*·*	S	*·*
A/Goa/046/2011	*·*	Q	*·*	T	*·*	*·*	*·*	*·*	*·*	V	*·*	*·*	*·*	*·*	*·*	*·*	*·*	*·*	*·*	*·*
A/Goa/047/2011	*·*	Q	*·*	*·*	*·*	*·*	*·*	*·*	*·*	V	I	*·*	*·*	*·*	*·*	*·*	*·*	*·*	S	*·*
A/Goa/048/2011	*·*	Q	R	*·*	*·*	*·*	*·*	*·*	*·*	V	L	*·*	*·*	*·*	*·*	*·*	*·*	*·*	S	*·*
A/Haryana/049/2011	*·*	*·*	*·*	*·*	I	*·*	*·*	*·*	*·*	V	*·*	*·*	*·*	V	*·*	*·*	*·*	*·*	S	*·*
A/Delhi/050/2011	*·*	*·*	*·*	*·*	I	*·*	*·*	*·*	*·*	V	*·*	*·*	*·*	*·*	*·*	*·*	*·*	*·*	S	*·*
A/Delhi/051/2011	*·*	*·*	*·*	*·*	I	*·*	*·*	*·*	T	V	*·*	*·*	*·*	*·*	*·*	*·*	*·*	*·*	S	*·*
A/Delhi/052/2011	*·*	*·*	*·*	*·*	I	*·*	*·*	*·*	T	V	*·*	*·*	*·*	*·*	*·*	*·*	*·*	*·*	S	*·*
A/Delhi/053/2011	*·*	*·*	*·*	*·*	I	*·*	*·*	*·*	T	V	*·*	*·*	*·*	*·*	*·*	*·*	*·*	*·*	S	*·*
A/Delhi/054/2011	*·*	*·*	*·*	*·*	I	*·*	*·*	*·*	T	V	L	*·*	*·*	*·*	*·*	*·*	*·*	*·*	S	*·*
A/Delhi/055/2011	*·*	*·*	*·*	*·*	I	*·*	*·*	*·*	*·*	V	*·*	*·*	*·*	*·*	*·*	*·*	*·*	*·*	S	*·*
A/Delhi/056/2011	*·*	*·*	*·*	*·*	I	*·*	*·*	*·*	*·*	V	*·*	*·*	*·*	*·*	*·*	*·*	*·*	*·*	S	*·*

A/Delhi/057/2012	*·*	*·*	*·*	*·*	I	*·*	*·*	*·*	*·*	V	*·*	*·*	*·*	*·*	*·*	*·*	*·*	*·*	S	*·*
A/Delhi/058/2012	*·*	*·*	*·*	*·*	I	*·*	*·*	*·*	*·*	V	*·*	*·*	*·*	*·*	*·*	*·*	*·*	*·*	S	*·*
A/Delhi/059/2012	*·*	*·*	*·*	*·*	I	*·*	*·*	*·*	*·*	V	*·*	*·*	*·*	*·*	*·*	*·*	*·*	*·*	S	*·*
A/Delhi/060/2012	*·*	*·*	*·*	*·*	I	*·*	*·*	*·*	*·*	V	*·*	*·*	*·*	*·*	*·*	*·*	*·*	*·*	S	*·*
A/Delhi/061/2012	*·*	*·*	*·*	*·*	I	*·*	*·*	*·*	*·*	V	*·*	*·*	*·*	*·*	*·*	*·*	*·*	*·*	S	*·*
A/Delhi/062/2012	*·*	*·*	*·*	*·*	I	*·*	*·*	*·*	*·*	V	*·*	*·*	*·*	*·*	*·*	*·*	*·*	*·*	S	*·*
A/Delhi/063/2012	*·*	*·*	*·*	*·*	I	*·*	*·*	*·*	*·*	V	*·*	*·*	*·*	*·*	*·*	*·*	*·*	*·*	S	*·*
A/Delhi/064/2012	*·*	*·*	*·*	*·*	I	*·*	*·*	*·*	*·*	V	*·*	*·*	*·*	*·*	*·*	*·*	*·*	*·*	S	*·*
A/Delhi/065/2012	*·*	*·*	*·*	*·*	I	*·*	*·*	*·*	*·*	V	*·*	*·*	*·*	*·*	*·*	*·*	*·*	*·*	S	*·*
A/Delhi/066/2012	*·*	*·*	*·*	*·*	I	*·*	*·*	*·*	*·*	V	*·*	*·*	*·*	*·*	*·*	*·*	*·*	*·*	S	*·*
A/Delhi/067/2012	*·*	*·*	*·*	*·*	I	*·*	*·*	*·*	*·*	V	*·*	*·*	*·*	*·*	*·*	*·*	*·*	*·*	S	*·*
A/Delhi/068/2012	*·*	*·*	*·*	*·*	I	*·*	*·*	*·*	*·*	V	*·*	*·*	*·*	*·*	*·*	*·*	*·*	*·*	S	*·*
A/Delhi/069/2012	*·*	*·*	*·*	*·*	I	*·*	*·*	*·*	*·*	V	*·*	*·*	*·*	*·*	*·*	*·*	*·*	*·*	S	*·*
A/Uttarakhand/070/2012	*·*	*·*	*·*	*·*	I	*·*	*·*	*·*	*·*	V	*·*	*·*	*·*	*·*	*·*	*·*	*·*	*·*	S	*·*
A/Goa/071/2012	*·*	*·*	*·*	*·*	I	*·*	*·*	*·*	*·*	V	*·*	*·*	*·*	*·*	*·*	*·*	*·*	*·*	S	*·*
A/Goa/072/2012	*·*	*·*	*·*	*·*	I	*·*	*·*	*·*	*·*	V	*·*	*·*	*·*	*·*	*·*	*·*	*·*	*·*	S	*·*
A/Goa/073/2012	*·*	*·*	*·*	*·*	I	*·*	*·*	*·*	*·*	V	*·*	*·*	*·*	*·*	*·*	*·*	*·*	*·*	S	*·*
A/Chhattisgarh/074/2012	*·*	*·*	*·*	*·*	I	*·*	*·*	*·*	*·*	V	*·*	*·*	*·*	*·*	*·*	*·*	*·*	*·*	S	*·*
A/Chhattisgarh/075/2012	*·*	*·*	*·*	*·*	I	*·*	*·*	*·*	*·*	V	*·*	*·*	*·*	*·*	*·*	*·*	*·*	*·*	S	*·*
A/Haryana/076/2012	*·*	*·*	*·*	*·*	I	*·*	*·*	*·*	*·*	V	*·*	*·*	*·*	*·*	*·*	*·*	*·*	*·*	S	*·*
A/Haryana/077/2012	*·*	*·*	*·*	*·*	I	*·*	*·*	*·*	*·*	V	*·*	Q	*·*	*·*	*·*	*·*	*·*	*·*	S	*·*
A/Haryana/078/2012	*·*	*·*	*·*	*·*	I	*·*	*·*	*·*	*·*	V	*·*	Q	*·*	*·*	*·*	*·*	*·*	*·*	S	*·*

A/Haryana/079/2013	*·*	Q	*·*	*·*	I	*·*	*·*	*·*	*·*	V	*·*	*·*	*·*	*·*	*·*	*·*	*·*	*·*	S	*·*
A/Haryana/080/2013	*·*	K	*·*	*·*	I	*·*	*·*	*·*	*·*	V	*·*	E	*·*	*·*	*·*	*·*	*·*	*·*	S	*·*
A/Haryana/081/2013	*·*	*·*	*·*	*·*	I	*·*	*·*	*·*	*·*	V	*·*	E	*·*	*·*	*·*	*·*	*·*	*·*	S	*·*
A/Uttarakhand/082/2013	*·*	*·*	*·*	*·*	I	*·*	*·*	*·*	*·*	V	*·*	E	*·*	*·*	*·*	*·*	*·*	*·*	S	*·*
A/Uttarakhand/083/2013	*·*	*·*	*·*	*·*	I	*·*	*·*	*·*	*·*	V	*·*	E	*·*	*·*	*·*	*·*	*·*	*·*	S	*·*
A/Jammu Kashmir/084/2013	*·*	*·*	*·*	*·*	I	*·*	*·*	*·*	*·*	V	*·*	*·*	*·*	*·*	*·*	*·*	*·*	P	S	*·*
A/Jammu Kashmir/085/2013	*·*	*·*	*·*	*·*	I	*·*	*·*	*·*	*·*	V	*·*	*·*	*·*	*·*	*·*	*·*	*·*	*·*	S	*·*
A/Delhi/086/2013	*·*	*·*	*·*	*·*	I	*·*	*·*	*·*	*·*	V	*·*	*·*	*·*	*·*	*·*	*·*	*·*	*·*	S	*·*
A/Delhi/087/2013	*·*	*·*	*·*	*·*	I	*·*	*·*	*·*	*·*	V	*·*	*·*	*·*	*·*	*·*	*·*	*·*	*·*	S	*·*
A/Delhi/088/2013	*·*	*·*	*·*	*·*	I	*·*	*·*	*·*	*·*	V	*·*	*·*	*·*	*·*	*·*	*·*	*·*	*·*	S	*·*
A/Delhi/089/2013	*·*	*·*	*·*	*·*	I	*·*	*·*	*·*	*·*	V	*·*	*·*	*·*	*·*	*·*	*·*	*·*	*·*	S	*·*
A/Uttar Pradesh/090/2013	*·*	*·*	*·*	*·*	I	*·*	*·*	*·*	*·*	V	*·*	*·*	*·*	*·*	*·*	*·*	*·*	*·*	S	*·*
A/Uttar Pradesh/091/2013	*·*	*·*	*·*	*·*	I	*·*	*·*	*·*	*·*	V	*·*	*·*	*·*	*·*	*·*	*·*	*·*	*·*	S	*·*

**Table 4:** Selection pressure analysis of NS1 protein and NEP of H1N1 pdm (09) virus using SLAC, FEL, REL, MEME, and FUBAR methods at (http://www.datamonkey.org).

Protein	Codon∗	SLAC dN-dS	SLAC *P* value	FEL dN-dS	FEL *P* value	REL dN-dS	REL Bayes factor	MEME *ω*+	MEME *P* value	FUBAR dN-dS	FUBAR post. pr.
NS1	55	17.614	0.131	73.794	0.056	3.898	3779.510	>100	0.069	3.151	0.989
	**108**	**5.330**	**0.459**	**19.133**	**0.208**	**3.149**	**11.971**	**>100**	**0.251**	**0.286**	**0.769**
	**123**	**5.399**	**0.447**	**24.424**	**0.142**	**3.558**	**22.722**	**>100**	**0.219**	**0.542**	**0.813**
	129	13.333	0.142	62.522	0.043	3.978	19238.300	>100	0.074	2.993	0.992
	**145**	**8.103**	**0.630**	**37.071**	**0.318**	**3.776**	**514.262**	**>100**	**0.328**	**1.285**	**0.924**
	**147**	**7.491**	**0.232**	**34.083**	**0.084**	**3.828**	**45.133**	**>100**	**0.101**	**1.081**	**0.880**
	**205**	**8.870**	**0.438**	**33.193**	**0.162**	**3.928**	**2940.990**	**>100**	**0.171**	**1.042**	**0.922**

NEP	49	6.729	0.457	134.502	0.158	0.260	2.013	>100	0.183	1.846	0.926

Significance value (SLAC *P* value = 0.5, FEL *P* value = 0.25, REL Bayes factor = 50, MEME *P* value = 0.1, FUBAR posterior probability = 0.9).

∗The sites found under positive selection by at least two methods are shown.

Sites present in NS1 host factor interaction domains are highlighted with bold font.

**Table 5:** Cluster specific amino acid changes were seen in all the sequences of cluster and were absent from sequences of all other clusters.

Clusters	Sites	Cluster specific amino acid changes
KOL 507 (2007, 2009)	13	D26, N53, C59, L85, V95, *N143* (99%), T145, N171, T209, F214, T216, T217, T226

KOL 596 (2007, 2009)	7	P3, R59, A60, S103, I106, A171, I226

KOL 989 (2007–2009)	19	V23, R41, A56, H59, K67, V82, T84, I95, L98, N101, E112, *M129* (80%), I144, V145, I171, K196, R224, A226, K229

NIV 6196 (2009–2013)	28	M6, F22, *N25* (99%), G26, L59, W67, S74, T76, R78, I81, T86, S91, *R108* (99%), *I111* (90%), I112, L119, *V123* (85%), V129, N139, Y171, I198, *N205 *(77%), C206, D207, S213, P215, E217, ∗220

Some cluster specific changes were seen in variable percentage of sequences in cluster and those absent from all other clusters are highlighted with italic font.

**Table 6:** Amino acid positions common between clusters.

Clusters	KOL 596	KOL 989	NIV 6196
KOL 507	18, 48, 67, 112, 125, 129, 197, 224, 229	21, 166, 178, 211	44, 101, 117
KOL 596	—	26, 44, 117, 217, 221	21, 84, 95, 145, 166, 178, 211
KOL 989	26, 44, 117, 217, 221	—	18, 48, 125, 197

Amino acid position seen in 100% to 65% of samples between a pair of clusters.

## Database

Nucleotide sequence data reported are available in the GenBank databases under the accession numbers “NCBI: KJ023091 to KJ023181.”
